# Feasibility of a cognitive behavioural group intervention to reduce fear of falling and associated avoidance of activity in community-living older people: a process evaluation

**DOI:** 10.1186/1472-6963-7-156

**Published:** 2007-09-27

**Authors:** Jolanda CM van Haastregt, GA Rixt Zijlstra, Erik van Rossum, Jacques ThM van Eijk, Luc P de Witte, Gertrudis IJM Kempen

**Affiliations:** 1 Maastricht University, Faculty of Health, Medicine and Life Sciences, School for Public Health and Primary Care, P.O. Box 616, 6200 MD, Maastricht, The Netherlands; 2 Professional University Zuyd, Faculty of Health and Technology, PO Box 550, 6400 AN, Heerlen, The Netherlands; 3 Institute for Rehabilitation Research, PO Box 192, 6430 AD, Hoensbroek, The Netherlands

## Abstract

**Background:**

Fear of falling and associated avoidance of activity are common among older people and may have negative consequences in terms of functional decline, quality of life and institutionalisation. We evaluated the effects of a cognitive behavioural group intervention to reduce fear of falling and associated avoidance of activity among older persons. This intervention showed favourable effects on fear of falling, avoidance of activity, daily activity, and several secondary outcomes. The aim of the present study is to assess the feasibility of this cognitive behavioural group intervention for participants and facilitators.

**Methods:**

The intervention consisted of eight weekly group sessions lasting two hours each and a booster session after six months. Self-administered questionnaires, registration forms and interviews were used to collect data from participants (n = 168) and facilitators (n = 6) on the extent to which the intervention was performed according to protocol, participant attendance, participant adherence, and participants' and facilitators' opinion of the intervention. Quantitative data from the questionnaires and registration forms were analysed by means of descriptive statistics. Qualitative data were categorised based on matching contents of the answers.

**Results:**

Facilitators reported no major protocol deviations. Twenty-six percent of the participants withdrew *before *the start of the programme. Of the persons who started the programme, 84% actually completed it. The participants reported their adherence as good, but facilitators had a less favourable opinion of this. The majority of participants still reported substantial benefits from the programme after six and twelve months of follow-up (71% and 61% respectively). Both participants and facilitators provided suggestions for improvement of the intervention.

**Conclusion:**

Results of this study show that the current cognitive behavioural group intervention is feasible for both participants and facilitators and fits in well with regular care. Minor refinement of the intervention, however, is warranted to further improve intervention effectiveness and efficiency. Based on these positive findings, we recommend implementing a refined version of this effective and feasible intervention in regular care.

**Trial registration:**

ISRCTN43792817

## Background

Fear of falling and associated activity restriction are common problems among older people. About 20 to 60 percent of older people living in the community are afraid to fall [1-5] and 20 to 55 percent report activity restriction due to fear of falling [5-9]. Fear of falling and associated avoidance of activity may have negative consequences in terms of functional decline, decreased quality of life and institutionalisation [2,10,11]. For this reason, Tennstedt and colleagues developed a cognitive behavioural group intervention primarily aimed at reducing fear of falling and avoidance of activity [12,13]. This intervention, called "A Matter of Balance", showed favourable effects on mobility, intended activity, social functioning, falls efficacy and the perceived ability to manage falls in the US [12]. Success of an intervention in the US is, however, no guarantee for its success in other countries. Therefore, we translated the protocol of this intervention into Dutch and made some adjustments to the protocol based on recommendations by experts, and experiences in the US and a pilot study [14]. Next, we carried out a randomised controlled trial (n = 540) to assess both short-term and long-term effects of "A Matter of Balance" in the Netherlands (ISRCTN43792817) [15,16]. Results of this trial showed that the intervention had favourable effects on all primary outcomes: fear of falling, avoidance of activity, and daily activity. Furthermore, favourable effects were also observed on several of the secondary outcomes [Zijlstra GAR, van Haastregt JCM, Ambergen T, van Rossum E, van Eijk JThM, Tennstedt SL, Kempen GIJM: Effectiveness of a cognitive behavioural group intervention on fear of falling and associated avoidance of activity in community-living older people: a randomised controlled trial. Submitted].

As has been widely acknowledged [17,18] a detailed process evaluation should be integral to the design of randomised controlled trials. Process evaluations may facilitate interpretation of outcomes, and recognition of strong and weak aspects of the intervention, and implementation of the intervention. In the present paper we discuss the results of a process evaluation which was performed alongside the trial [15,16]. The aim of the present process evaluation is to study the feasibility of the intervention by assessing: a) the extent to which facilitators reported that the intervention was performed according to protocol; b) participant attendance; c) participant adherence; and d) participants' and facilitators' opinion of the intervention.

## Methods

### Study design and population

The current process evaluation is a descriptive study with longitudinal elements, in which quantitative and qualitative data were gathered. Our study population consisted of 280 older persons who received a cognitive behavioural intervention [15] and six facilitators who conducted this intervention. All 280 participants were community-living, aged 70 years or older and had reported at least some fear of falling and related activity restriction in a screening questionnaire [15,16]. This questionnaire was sent to a random sample of 7,431 older persons living independently in either Heerlen or Maastricht, in the south of the Netherlands [15,16]. Time period between receiving the screening questionnaire and starting the intervention was about 3 months. The facilitators were community nurses specialised in geriatric care and employed with two local homecare organisations who received two days' training before conducting the intervention. Details about study design and population are reported elsewhere [15,16]

### Intervention

The intervention is a cognitive behavioural group programme developed to reduce fear of falling and to promote activity among older persons living in the community. The intervention consists of eight weekly sessions lasting 120 minutes and a booster session at six months after the end of the intervention lasting 135 minutes. The first session is conducted by two facilitators; the next seven sessions and the booster session by one. The second facilitator serves as a substitute for the first facilitator in case of his or her absence.

The intervention employs the following strategies to reduce fear of falling and activity restriction: a) restructuring misconceptions to promote the view that the risk of falls and fear of falling are controllable; b) setting realistic goals for increasing activity; c) changing the environment to reduce the risk of falls; and d) promoting physical exercise to increase strength and balance. The themes of the eight sessions are presented in Table 1 and are described elsewhere in more detail [14].

**Table 1:** 

Session themes of the Dutch version* of "A Matter of Balance"	
1.	Introduction to the programme
2.	Exploring thoughts and concerns about falling
3.	Exercise and fall prevention
4.	Assertiveness and fall prevention
5.	Managing concerns about falling
6.	Recognising fall-ty habits
7.	Recognising fall hazards in the home and community
8.	Practicing no fall-ty habits

9.	Booster session

* Note: a more detailed description is presented elsewhere [14,15]

A variety of didactic techniques is used during the sessions, including lectures, videos, group discussions, mutual problem-solving, physical exercises and skill development. The participants are given homework at the end of each session. This homework includes reading informative hand-outs, challenging concerns about falling on pre-structured forms, filling in home safety checklists and filling in personal action planners. In addition, facilitators encourage participants to practise the physical exercises at home both during and after the programme. The exercises are taught during the sessions and are described in detail in illustrated hand-outs. More information about the intervention protocol can be obtained from the authors [14].

Between February 2003 and May 2004, twenty groups received the intervention provided by the six facilitators. Each group consisted of approximately ten participants. Participants who were unable to come to the programme location independently were offered free transportation by taxi.

### Data collection

To study the feasibility of the intervention the following process outcomes were assessed: the extent to which facilitators reported that the intervention was performed according to protocol, participant attendance, participant adherence, and participants' and facilitators' opinion of the intervention [Table 2]. Data were collected from participants by means of self-administered questionnaires and short interviews by telephone. Participants received a questionnaire directly after the last session of the programme (FU1) and at six (FU2) and twelve (FU3) months after the programme. The questionnaires were sent only to those who had completed the programme (i.e. persons who had not withdrawn during the eight intervention sessions). Registration forms and self-administered questionnaires were used to collect data from the facilitators. The facilitators were asked to fill in a registration form after each session including questions about: time spent on performing the intervention; performance according to protocol; nature of and reasons for protocol deviations; adherence of the group during the session; and strong and weak aspects of the session. In addition they received an overall evaluative questionnaire directly after the end of the programme and after the booster session. In addition, the facilitators discussed and explained their written reports in two group meetings to conclude and evaluate the intervention. The following background characteristics were gathered before randomisation by means of self-administered questionnaires: age, gender, living alone or not, educational level, cognitive status (Dutch version of the Telephone Interview for cognitive status (TICS), use of walking aids, perceived general health (item one of the MOS SF-20), fear of falling (Are you afraid of falling?; 1 = never to 5 = very often), and avoidance of activity due to fear of falling (Do you avoid certain activities due to fear of falling?; 1 = never to 5 = always) [15]. All data were gathered in the period between February 2003 and June 2004. The intervention and measurement instruments were pre-tested in a pilot study among 11 persons who met the inclusion criteria [14].

**Table 2:** Outcome measures of the process evaluation

Variables	BDI	FU1	FU2	FU3
*Performance intervention according to protocol*				
duration of the sessions	R^f^	-	-	-
deviations from protocol	R^f^	-	-	-
*Participant attendance*				
reasons for refusal before the start of the intervention	TI^p^	-	-	-
number of sessions visited by each participant	R^f^	-	R^f^	-
reasons for stopping during the intervention period	TI^p^	-	TI^p^	-
*Participant adherence*				
adherence to homework assignments	-	Q^p^/Q^f^	-	-
adherence to physical exercise	-	Q^p^/Q^f^	Q^p^	Q^p^
*Opinion about intervention*				
overall opinion of the intervention (grade)	-	Q^p^/Q^f^	-	-
opinion of the facilitators (grade)	-	Q^p^/Q^f^	-	-
benefits experienced by participants		Q^p^/Q^f^	Q^p^	Q^p^
strong and weak aspects of the intervention	-	Q^p^/Q^f^	-	-
suggestions for improvement	-	Q^p^/Q^f^	Q^f^	

BDI = before or during intervention; FU1 = direct follow-up (directly after the intervention);

FU2 = 6-month follow-up; FU3 = 12-month follow-up; R = registration form filled in after each session; Q = questionnaire; TI = telephone interview.

Data collected from: ^f ^= facilitator; ^p ^= participant.

### Data analysis

Quantitative data from the questionnaires and registration forms were analysed by means of descriptive statistics. Qualitative data from the questionnaires and registration forms (i.e. the answers to open questions) were categorised until themes and patterns in the answers emerged. The discussions of the facilitators during the group meetings were recorded on audiotape and transcribed. Relevant information resulting from these two meetings was used to facilitate the interpretation of the results of the questionnaires and registration forms filled in by the facilitators.

### Ethical considerations

This study, which is part of a larger study, was approved by the Medical Ethics Committee of Maastricht University/University Hospital Maastricht. All participants signed an informed consent form.

## Results

### Response

**Figure 1:**
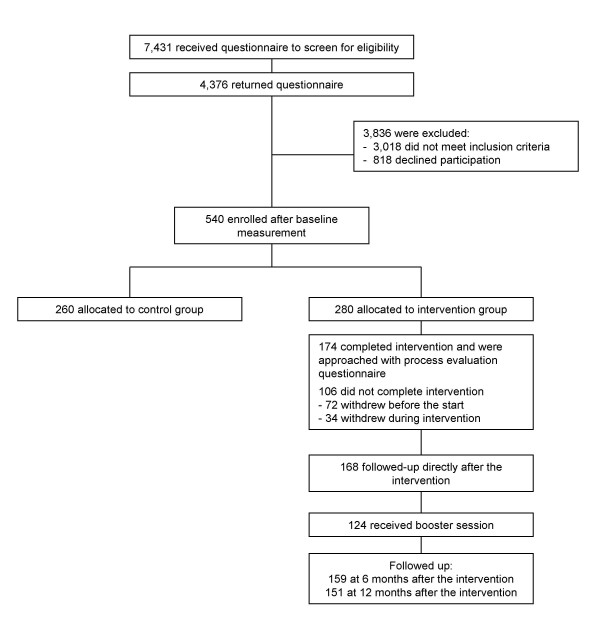
Flow chart process evaluation participants.

A total of 174 persons (74%) had completed the programme and received the first evaluation questionnaire immediately after the programme (Figure 1). Six questionnaires were not returned, resulting in a response of 97% (n = 168). Of the 161 persons who did not withdraw in the period between the end of the programme and the booster session after six months, 159 (99%) filled in the second questionnaire at FU2 and 151 (94%), the third at FU3. Table 3 shows the characteristics of the 174 persons who completed the intervention and the characteristics of the 106 persons who did not complete the intervention. Overall the persons completing the intervention seem to be less frail, less afraid of falling and less avoidant than the persons who did not complete the intervention.

**Table 3:** General characteristics of participants

General characteristics	Participants who completed the intervention		Participants who did not complete the intervention	
Values are number (percentage) unless stated otherwise	N = 174		N = 106	
Mean age (standard deviation)	77	(4.5)	78	(4.8)
Female	121	(70)	77	(73)
Living alone	100	(58)	57	(54)
≤ Lower secondary education	111	(64)	74	(70)
Mean cognitive status (standard deviation)*	32	(3.5)	31	(3.7)
Using walking aids	61	(35)	45	(43)
Fair or bad perceived health	118	(68)	79	(75)
At least one fall in previous 6 months	96	(56)	79	(57)
Fear of falling				
sometimes	108	(62)	52	(49)
often	40	(23)	33	(31)
very often	26	(15)	21	(20)
Avoidance of activity				
sometimes	117	(67)	51	(48)
often	36	(21)	40	(38)
very often	21	(12)	15	(14)

* scores range from 16 to 38; a lower score indicates a better cognitive functioning

Facilitators filled in registration forms for all but one of the 160 programme sessions (20 groups × 8 sessions) and for all the booster sessions (n = 20). All facilitators filled in the evaluation questionnaires after the end of the programme and after the booster session, and all facilitators participated in at least one of the two group meetings.

### Implementation of intervention according to protocol

Mean duration of the sessions was 123 minutes for the programme sessions and 135 minutes for the booster session. A total of 160 programme sessions (20 groups × 8 sessions) were conducted. Only 19 booster sessions were held, rather than the prescribed 20, because the sessions of two small groups were combined into one. On the registration forms filled in after each session (n = 178; 159 programme session forms and 19 booster session forms), the facilitators reported carrying out 88% (n = 156) of all sessions according to protocol. Protocol deviations were reported for 12% of the sessions (n = 22, i.e. an average of one session for every group). In all cases, this involved skipping entirely or in part one of the activities of a session. Since each session consists of a number of different activities, with many activities being repeated in multiple sessions, skipping one activity in a session was considered to be a minor protocol deviation. The facilitators reported no major deviations from protocol.

### Participant attendance

Of the eight intervention sessions, 162 participants (58%) attended at least five sessions and 118 participants (42%) attended less than five sessions. Seventy-two (26%) of the 280 persons allocated to the intervention group withdrew *before *the start of the programme. The main reasons for withdrawal were health problems (n = 25), considering the intervention to be inappropriate for their needs (n = 10) and being too busy with other activities (n = 10). Thirty-four persons (12%) withdrew *during *the programme, after attending an average of 1.6 sessions. The main reasons for withdrawal in this group were health problems (n = 12) and finding the intervention to be inappropriate for their needs (n = 8).

A total number of 174 persons (62%) completed the programme, attending 6.8 sessions on average. Thirteen persons from this group withdrew in the period between the end of the programme and the booster session, mostly for reasons related to health (n = 7). Of the remaining 161 persons, 124 actually attended the booster session (77%). On average, 14 persons were allocated to a group. Of these, an average of ten actually started the programme and nine completed it. An average of six persons participated in the booster session.

### Participant adherence

Directly after the end of the programme, participants were asked how often they did the homework assigned to them by the facilitator. Of the 167 persons who answered this question, 12 (7%) reported they did their homework never or rarely, 30 (18%) that they sometimes did, and 125 (75%) said they usually or always did their homework. The participants who reported that they sometimes, usually or always did their homework spent an average of 29 minutes (s.d. = 22) on it per session (with a range from 10 to 120 minutes). The facilitators were also asked to assess the adherence to homework for each of their groups. According to the facilitators, in six of the twenty groups the majority of participants did their homework. In eight groups, this applied to about half of the participants, while in six groups only a minority of the participants did their homework. The general quality of the homework made by participants was considered sufficient in 13 groups and insufficient in seven groups.

Directly after the intervention period, the booster session and six months after that again, the participants were asked how often they had done the exercises in the previous period and how much time they had spent doing them. Table 4 shows that the frequency of doing the exercises declined considerably in the period between the end of the programme and the six-month follow-up. However, the exercise frequency remained fairly stable between six and twelve months after the programme. Time spent on the exercises also remained stable during the follow-up period. The facilitators were asked to assess, for each of their groups, the adherence to the physical exercises. According to the facilitators, in eight of the twenty groups most participants did do the exercises at home. In seven groups, about half of the participants did the exercises at home, while in five groups only a minority of the participants did so.

**Table 4:** Adherence to physical exercises according to the participants

	During the programme		After 6 months		After 12 months	
	n = 168		n = 159		n = 151	
	N	%	N	%	N	%
How often did you do the physical exercises?						
• never	15	9	22	14	22	15
• less than once a week	13	8	52	33	55	36
• once a week	40	24	39	25	34	23
• more than once a week	100	60	46	29	40	27
						
How much time did you spend (on average) on your physical exercises each time you did them?*						
• < 10 minutes	65	43	67	50	60	47
• 10 to 20 minutes	59	39	51	38	49	38
• 20 to 30 minutes	22	14	13	10	15	12
• 30 minutes or more	7	5	4	3	4	3

* only filled in by persons who (still) did the exercises; there were 2 missing values at 6 months and 1 at 12 months after the intervention

### Opinion of the intervention

#### Overall opinion of the intervention and facilitators

Directly after the programme, participants and facilitators were asked to give the programme a report mark ranging from 1 to 10, where 1 is the most negative score and 10 the most positive. Participants gave a mean report mark of 8 (range = 5–10, n = 168), facilitators an average of 7.5 (range = 7–8, n = 20 groups assessed by 6 facilitators). Participants had a very positive opinion of the facilitators; 98% percent considered the facilitators to be good or very good. The six facilitators themselves also had a fairly favourable opinion of their own role. In 14 groups, they qualified their functioning as good and in six groups as sufficient.

#### Benefits reported by participants

Directly after the programme, participants were asked whether they felt they had benefited from the programme regarding 12 specific topics addressed during the programme (Table 5). The percentage of participants who felt they benefited ranged from 88% for the topic "I behave more safely" to 46% for "I avoid fewer activities". In addition, at six and twelve months follow-up, participants answered a general question regarding the overall benefit they experienced from the programme in the preceding six months. At six months follow-up, 71% of the participants said they had benefited much or very much from the programme in the past 6 months; at 12 months follow-up, this percentage had fallen somewhat (61%).

**Table 5:** Benefits experienced from the programme by participants, as judged by participants and facilitators

Due to following the programme....	Participants (n = 174)		Facilitators (n = 6)	
	
	Yes, I agree		Yes, this is the case for the majority of participants in this group (n = 20 groups)*	
	N	%	N	%

I behave more safely	147	88%	9	45%
my self-confidence has increased	134	80%	10	50%
I am able to change negative thoughts into helpful thoughts	138	79%	6	30%
I know better how to reduce the negative consequences of falling	133	79%	11	55%
I became more physically active	132	79%	12	60%
I behave more assertively	129	77%	11	55%
I am less concerned to fall	124	74%	5	26%
my risk of falling is reduced	111	66%	7	35%
my home environment became safer	102	61%	6	30%
my balance increased	103	59%	5	25%
my muscle strength improved	82	49%	10	50%
I avoid fewer activities	76	46%	4	21%

* Facilitators were asked "Do you think that the participants..." followed by the topics mentioned in the first column.

Facilitators were also asked to assess the benefits of the programme for each of their groups (they were asked "Do you think the participants...." followed by the items mentioned in the first column of Table 5). In general, the facilitators were less optimistic than participants about the programme benefits. The facilitators were most positive about the benefits in the field of physical activity. They assessed that the majority of participants had become more physically active in 12 of the 20 groups. The influence of the programme on activity restriction was regarded least positively (only 4 of the 20 groups benefited).

#### Strong and weak aspects of the intervention

Both participants and facilitators were asked to name strong and weak aspects of the programme (open questions). The strong aspects mentioned most frequently by participants (n = 168) were: the information provided by the facilitators (n = 65), the role of the facilitators e.g. their enthusiasm and clarity (n = 63), the physical exercises (n = 47), the interaction with other participants e.g. socialising and learning from each other (n = 43), and raising awareness about home safety and safe behaviour (n = 16). The strongest aspects of the programme according to the facilitators were: the interaction between participants (e.g. showing commitment, learning from each other and helping each other), the physical exercises, raising awareness about home safety and safe behaviour, and the promotion of assertive behaviour.

The weak aspects mentioned most frequently by participants were: homework was too much and/or too difficult (n = 25), too much repetition of topics (n = 10), the first two sessions were boring (n = 8) and too much chattering during the sessions by other participants (n = 6). The majority of participants, however, could not come up with any weak aspects (n = 94). Weak aspects reported by the facilitators were mainly related to the homework assignments, which were considered too difficult for most participants. According to the facilitators, participants often failed to do their homework as intended. In addition, the facilitators mentioned that participants appeared to have difficulty with abstract thinking (which is needed in the process of cognitive restructuring) and with reproducing the topics discussed in previous sessions.

#### Suggestions for improvement

The majority of participants made no suggestions for improvement (n = 91). Seventy-three participants did have suggestions, these mostly being: simpler homework (n = 19), more physical exercises during the sessions (n = 14) and additional (booster) sessions (n = 8). The main suggestions made by facilitators were: simpler homework, a minimum of 8 and a maximum of 10 participants per group, fewer topics to be discussed in each session or an increase in the number of sessions, and a more targeted selection of participants. The facilitators considered the intervention especially appropriate for people who feel seriously restricted by their fear of falling, who are motivated to tackle this problem, who are functioning quite well cognitively and who are somewhat used to reading. The facilitators considered the intervention less appropriate for people with strongly impaired vision, a hearing impairment, psychiatric problems like depression, or serious physical impairments.

## Discussion

In this paper we assessed the feasibility of a cognitive behavioural intervention aimed at reducing fear of falling and associated avoidance of activity among older persons living in the community. As reported by the facilitators there were no major protocol deviations. More than a quarter (n = 72) of the 280 participants never started the programme, mainly due to health problems. Of the 208 persons who did start, 84% completed the programme. The participants reported their adherence as good, but facilitators had a less favourable opinion of this. Both participants and facilitators were positive about the programme and the majority of participants reported benefits from it. Both participants and facilitators provided suggestions for improvement of the intervention.

When comparing our results with the results of Tennstedt and colleagues who originally developed the programme, the pattern of withdrawals appears to differ [12]. Tennstedt reported that 16% attended no sessions at all while in our study 26% attended no sessions at all. The higher percentage of persons who attended no sessions at all in our study may be explained by differences in recruitment strategies and characteristics of the participants. Tennstedt et al. recruited participants through self-response to posted notices and individual referrals by housing managers, social workers and case managers and eligibility of their participants was determined during a home visit [12]. In our study participants were recruited by means of a short screening questionnaire which was sent to a random sample of older persons [15]. A personal intake procedure as used by Tennstedt would probably reduce the percentage of early withdrawal because the eligibility criteria can be checked more closely and tailor-made information about the programme can be provided to potential participants. In addition, we included community-living older persons and conducted the intervention at a location somewhere in the vicinity of the participants, while Tennstedt and colleagues recruited persons from senior housing sites and conducted the intervention at these senior housing sites. The latter may have resulted in lower barriers to attend the group meetings.

A possible limitation of our study is the risk that participants may have given social desirable answers to our questions. We tried to reduce this tendency by using self-administered questionnaires and by making it clear to participants that the facilitators would not be informed about their individual answers and that their answers would not affect any care needs now or in the future. However, the reasons for withdrawal from the programme were collected by means of telephone interviews, making it perhaps more difficult for participants to report discontent with the programme as the main reason for withdrawal. Strengths of our study are that we collected data from both participants and facilitators by using different methods and that we, in order to avoid bias, analysed the data of the process evaluation before analysing the data of the effect evaluation.

This process evaluation provides insight into the strong and weaker aspects of the intervention. A strong aspect of the intervention is that the majority of participants who actually started the programme, considered it to be beneficial. Another strong aspect is that the six geriatric nurses participating in our study were very capable of conducting the programme and all said that they would continue conducting the programme after the end of the trial, if given the opportunity. The following aspects of the intervention need to be improved. First, the homework assignments were found to be too complicated for part of the participants in our research population. Second, participant adherence to the physical exercises seemed not optimal, although restricted adherence to home exercises seems common among community-dwelling older people participating in fall prevention interventions [19,20]. Third, about a quarter of the selected participants withdrew before the programme even started, mainly because of health problems. This suggests that we did not completely succeed in: a) selecting the most appropriate population; b) motivating persons who were doubtful about the potential benefits of the programme; and/or c) meeting the conditions under which even the most frail participants were willing and able to participate (although we offered free transportation to the programme location for those who needed this).

## Conclusion

Although we think that some aspects of the intervention could be refined, the results of the present study show that this effective intervention is feasible for both participants and facilitators and fits in well with regular care. Based on the results of this process evaluation, we recommend adapting the intervention on the following aspects. First, in order to tailor the intervention more to the capacities and skills of the target population, the homework assignments should be simplified to some extent. Second, measures should be taken to increase adherence to the physical exercises. This might be achieved by paying more attention during the programme to factors that may impede the participants from doing the exercises at home and clearly reinforcing the desired behaviour by giving compliments to those participants who show a good adherence. Long-term adherence to the exercises may be improved by adding incentives such as pre-scheduled motivational phone calls. Third, we recommend an individual intake interview for participation in the intervention. In our opinion, such intake interviews should be done by the programme facilitator (a geriatric nurse). Potential participants could be referred for an intake interview by health care professionals such as general practitioners, community nurses and geriatricians. In addition, potential participants could be informed about the programme through announcements in local media. Important factors during the intake interviews include: a) checking whether the potential participant fulfils the eligibility criteria; b) providing clear information about the content of the programme; c) motivating the potential participant; and d) paying attention to factors which may impede participation. We expect that such a procedure would increase the efficiency of the intervention by reducing the number of withdrawals before and during the programme. We recommend to implement the adapted version of the intervention in regular care. For persons with serious health problems for whom it is too burdensome to participate in a group intervention, we recommend developing an individualized in-home version of the intervention.

## Competing interests

The author(s) declare that they have no competing interests.

## Authors' contributions

All authors contributed to the development of the design of this study. JvH drafted the manuscript with input from the other authors. All authors read and approved the final manuscript.

## Pre-publication history

The pre-publication history for this paper can be accessed here:


